# Variations in VOCs Emissions and Their O_3_ and SOA Formation Potential among Different Ages of Plant Foliage

**DOI:** 10.3390/toxics11080645

**Published:** 2023-07-25

**Authors:** Baowen Zhang, Lili Qiao, Huijuan Han, Wenxia Xie, Lingyu Li

**Affiliations:** College of Environmental Sciences and Engineering, Carbon Neutrality and Eco-Environmental Technology Innovation Center of Qingdao, Qingdao University, Qingdao 266071, China; zhangbaowen28@163.com (B.Z.); 13210158173@163.com (L.Q.); hanhuijuan2022@163.com (H.H.)

**Keywords:** biogenic volatile organic compound, ozone, secondary organic aerosol, leaf age

## Abstract

Volatile organic compounds (VOCs) emitted by plant foliage play an important role in ozone (O_3_) and secondary organic aerosol (SOA) formation. Their emissions can be influenced by the leaf age. We explored the VOCs emissions and their effects on the formation of O_3_ and SOA from plant foliage in different ages. VOCs emissions from the young, mature, and senescent leaves of *Ginkgo biloba*, *Ligustrum lucidum*, and *Forsythia suspensa* were measured using the dynamic enclosure system and the TD–GC–MS technique. Based on the emission rates of quantified compounds, their potential to form O_3_ and SOA was estimated. Results showed that there were significant differences in the VOCs emission rate and their composition among leaves in different ages. The emission rate of the total VOCs by young leaves was the highest, while the lowest by senescent leaves. Monoterpenes were the dominant VOCs category, and isoprene emission had the lowest contribution for the leaves at each age. With increasing leaf age, the proportion of monoterpenes emission increased, and the proportion of sesquiterpenes decreased. The variations of isoprene and other VOCs were different. The potentials of total VOCs, isoprene, monoterpenes, sesquiterpenes, and other VOCs to form O_3_ (OFP) and SOA (SOAP) varied significantly among leaves at different ages. The total OFP and SOAP were the highest by young leaves, while the lowest by senescent leaves. With increasing leaf age, the contribution of monoterpenes to OFP and SOAP also increased, while that of sesquiterpenes decreased. Our study will provide support for the more accurate parameterization of the emission model and help to understand the VOCs emissions and study the precise prevention and control of complex air pollution at different times.

## 1. Introduction

Fine particulate matter (PM_2.5_) and ozone (O_3_) are currently important atmospheric pollutants affecting air quality in urban and regional areas of China [1,2,3,4]. Volatile organic compounds (VOCs) are important precursors of O_3_ and secondary organic aerosols (SOA, the important component of PM_2.5_) formation, which play an important role in the formation of regional complex air pollution and also have an important impact on regional and global climates [4,5,6]. VOCs in the air come from anthropogenic and biogenic emissions. In China, many control measures have been established to alleviate the anthropogenic VOC emission, resulting in their decrease in recent years [7]. Whereas, biogenic VOCs emissions kept increasing because of the expanding vegetation cover [8,9]. Globally, BVOCs emissions are as high as 1000 Tg/year, accounting for 70–90% of the total VOCs emissions, far more than the VOCs released by anthropogenic activities [10,11]. At the same time, biogenic VOCs, especially terpenes, have high chemical activity, which can be converted into secondary organic pollutants for a short time (a few minutes to 2–3 h) in the atmosphere [12,13]. In summer 2018, biogenic VOCs emissions contributed 16.8% in daily maximum 8 h O_3_ and 73.15% in SOA over China; the contribution was much higher in some areas [14]. Therefore, understanding the emission characteristics of biogenic VOCs is of great significance to study the formation mechanism and prevention and control of complex air pollution in China.

VOCs emission from plants depends not only on environmental conditions and abiotic factors, but also on individual development [15,16,17,18]. The leaf at different ages has different VOCs emission. Although many studies have been conducted on VOCs emission from mature leaves of plants [19,20,21], there are relatively few studies on plant foliage at different ages. In addition, most of the studied plants were trees and crops, and there were few studies for shrubs. For example, Mozaffar et al. compared the VOC emission from young, mature, and senescent leaves of maize [22]. Portillo-Estrada and Holopainen recorded the VOC emission in senescent leaves of Populus spp and Betula pendula, respectively [23]. Bison et al. investigated the characteristics of VOCs emission from young and mature leaves of *Croton floribundus* [24]. Meanwhile, VOCs emission observation is mainly focused on isoprene and some monoterpenes [25], and there are few studies on other VOCs. Studies have shown that sesquiterpenes and aromatic hydrocarbons are also important components of plant VOC emission [26].

In urban areas, VOCs can contribute more to the O_3_ enhancement. It is significant to have a comprehensive knowledge on the VOC emissions by urban green plants. *Ginkgo biloba*, *Ligustrum lucidum*, and *Forsythia suspensa* are important plants for urban greening in northern China (from the Recommended List of Tree Species for Afforestation in Shandong Province). In this study, VOC emissions by leaves at different leaf ages (young leaves, mature leaves, and senescent leaves) for the three plant species were observed. The variations in VOCs emission rates and their composition among different leaf ages were investigated. Their potential to form O_3_ and SOA were estimated. This study will provide basic data for studying the biogenic VOCs emissions and the precise prevention and control of complex air pollution in China at different times.

## 2. Methods

### 2.1. Experimental Design

The potted plants were used in this study. After the plants were purchased, they were allowed to acclimate to the environment for one month. During this period, they were fully exposed to sunlight and irrigated every two to three days to maintain sufficient soil moisture. Three parallel individuals were set for each plant species. They were grown in plastic pots (3.5 L in volume, 19 cm in caliber, 18 cm in height, and 13.5 cm in bottom diameter) with commercial soil (including peat soil, vermiculite, coconut residuum, organic matter, and perlite). All the plants were 40–60 cm high.

VOCs emission samples of *Ginkgo biloba*, *Ligustrum lucidum*, and *Forsythia suspensa* were collected in mid-April (for young leaves), mid-July (for mature leaves), and late September (for senescent leaves).

### 2.2. BVOCs Measurement

VOCs were collected using the dynamic enclosure system [27]. The sampling was conducted during 8:00–17:00. Then, 19 L (50 cm × 60 cm) or 114 L (80 cm × 110 cm) Teflon bags (Welch Fluorocarbon, Inc., USA), which were almost 100% transparent to photosynthetically active radiation (PAR), were used to enclose the plant. The 19 L Teflon bag was used only when measuring emissions of young leaves of *Ligustrum lucidum*. A Teflon bag is rectangular with one open end. When enclosing, the potted plant was on a Teflon round platform, the bag enclosed the potted plant, and its open end was sealed tightly with a rubber band circling around the platform. There were three interfaces on the platform connecting two 0.25 inch o.d. Teflon tubes and one temperature sensor reaching into the bag. The vacuum pump (DOA-P504-BN, GAST^®^, Chicago, IL, USA) and mass flow controller (MFC) (CS300, SevenStar, Beijing, China) were used to pump the purified air (the ambient air after removing VOCs and O_3_ by passing through the active carbon (100 mesh, SCR^®^, Shanghai, China)) into the Teflon bag through one tube at the constant flow rate of 10 and 20 L/min for 19 L and 114 L bags, respectively. At the same time, the gas in the sampling bag flowed out through another tube at the same flow rate, so that the gas in the sampling bag kept circulating. The system was equilibrated for 1–2 h after enclosure. Then, the gases in the chamber were collected onto adsorption tubes (filled with Tenax TA and Carbograph 5TD) using an air sampling pump (Gilian Gilair Plus, Sensidyne, St. Petersburg, FL, USA). The sampling flow rate was 200 mL/min, and the sampling volume was 6 L. For each individual plant, three parallel emission samples and one blank sample were collected at each leaf age. Blank samples were collected by the same method, and the bag enclosed the pot with the same volume of soil and without plants. During the experiment, the temperature and PAR were continuously (per 5 s) monitored by the temperature sensor (109, Campbell Scientific, Inc., Logan, UT, USA) and PAR sensor (LI-190R, LI-COR Corporation, Lincoln, NE, USA). The temperature sensor was placed inside the bag, and the PAR sensor was placed outside. After sampling, the adsorption tubes were placed in the refrigerator at 4 °C, and analysis was completed within one month. At the end of sampling, the closed leaves were dried at 75 °C until constant weight. For young and mature leaves, five leaves with different sizes were selected as representatives to estimate their dry weight and leaf area, and the number of leaves with corresponding size was obtained, and then the total dry weight of closed leaves was estimated according to the ratio of dry weight to leaf area. For senescent leaves, all leaves were removed to obtain the dry weight. The dry weight of the leaves was measured by an electronic balance.

VOCs samples were analyzed using the thermal desorption–gas chromatography–mass spectrometry (TD–GC–MS; TD, ATD II-26, Acrichi Inc., Beijing, China; GC–MS, 7890A-5975C, Agilent Technologies, Santa Clara, CA, USA). The temperature of the injection valve was 150 °C, the temperature of thermal desorption was 290 °C, the desorption duration was 10 min, the temperature of the cold trap was −30 °C, and the carrier gas was nitrogen. The working conditions of GC were as follows: the temperature of the sample inlet was 250 °C, the Agilent DB-5 capillary column (30 m × 0.25 mm ID × 0.25 μm) was used, the carrier gas flow rate was 1.2 mL/min, and the split ratio was 1:5; the initial temperature for the programmed heating was 35 °C and maintained for 2 min, then it was increased to 260 °C at the speed of 10 °C/min and maintained for 21 min; lastly, the temperature was increased to 280 °C and maintained for 2 min. MS operating conditions: the EI source ion energy was 70 eV, the ion source temperature was 200 °C, the interface temperature was 250 °C, and the filament current was 150 μA.

The quantified compounds included isoprene, 14 monoterpenes, 6 sesquiterpenes, and 42 other VOCs (including 21 alkanes, 4 alkenes, and 17 aromatics) (Table S1). They were quantified by methods of response factor (RF) and external standard using the photochemical assessment monitoring station (PAMS) mixed standards (LINDE, Stewartsville, NJ, USA) and terpene mixed standards (Apel-riemer environmental, Miami, FL, USA) with the concentration of 1 ppm. RFs were used when the relative standard deviation was ≤20%; otherwise, the external standard was used which has the correlation coefficient of >0.99 for the standard curves. The detection limits for various compounds were in the range of 0.03–0.99 μg/m^3^ when the sample volume was 6 L, and the precision was 4–15%, as listed in Table S1 in detail.

The emission rates of VOCs were calculated according to Equation (1).

(1) $${\mathrm{EF}}_{i}=\frac{{F\;\times \;C}_{i}}{M}$$

where EF_i_ is the emission rate of VOCs species i, μg/(g·h); C_i_ is the mass concentration of VOCs species i of the sample after subtracting the blank, μg/L; M is the dry mass of the enclosed leaves, g; and F is the flow rate of purified air, L/h.

Referring to Guenther et al. [28], the actual emission rates of each VOCs component were converted to the standard ones under the standard conditions of temperature = 30 °C and PAR = 1000 μmol/(m^2^·s). In this study, the relative standard deviations of the VOC emissions among the three parallel individuals of each plant species were in the range of 2–23%.

### 2.3. Calculation of O_3_ and SOA Formation Potentials

O_3_ formation potential (OFP) reflects the maximum potential contribution of each VOCs to O_3_ formation under the optimal reaction conditions. OFP was calculated based on VOCs emission rate and their maximum incremental reaction (MIR). MIR is suitable for the areas where O_3_ is limited by VOCs, and most urban areas in China are VOCs-limited conditions; therefore, the application of MIR is suitable for the study of O_3_ formation in most areas of China. However, for some areas where O_3_ is NO_x_-limited, further consideration is needed. The OFP calculation formula is as follows [29]:

(2) $${\mathrm{OFP}}_{i}{=\mathrm{EF}}_{i}{\;\times \;\mathrm{MIR}}_{i}$$

where OFP_i_ is the OFP value of VOCs species i, μg/(g·h); EF_i_ is the emissions rate of VOCs species i, μg/(g·h); and MIR_i_ is the maximum incremental reactivity of VOCs species i, g O_3_/g VOCs (Table S1).

The potential of different VOCs to form SOA (SOAP) can be calculated by fractional aerosol coefficient (FAC), and the formula is as follows [30,31]:

(3) $${\mathrm{SOAP}}_{i}{=\mathrm{EF}}_{i}{\;\times \;\mathrm{FAC}}_{i}$$

where SOAP_i_ is the SOAP value of VOCs species i, μg/(g·h); EF_i_ is the emissions rate of VOCs species i, μg/(g·h); and FAC is the aerosol formation coefficient of the VOCs component i, % (Table S1).

### 2.4. Statistical Analysis

Using IBM SPSS Statistics 25 statistical software, one-way analysis of variance (ANOVA) and the least significant difference (LSD) method were used to evaluate the significance levels (*p* < 0.05) of differences in the VOCs emission rate, OFP, and SOAP. Means that do not share the same letter are significantly different. All graphs were generated by Origin 2018 software.

## 3. Results and Discussion

### 3.1. VOC Emissions Rate at Different Leaf Ages

Plant foliages at different ages have different characterizations. The young leaves of the studied plant were tender green. With the increase of leaf age, the color of the mature leaves gradually changes to dark green. Entering the senescence stage, the color of *Ginkgo biloba* and *Ligustrum lucidum* was deeper, the edge of *Ginkgo biloba* appears yellow, and *Forsythia suspensa* turns to red. The dry weight of leaves at different ages also changed. The weight of young leaves ranged from 2.42 g to 5.21 g. With the growth of the plants, the weight of the leaves increased, and the dry weight of the mature leaves ranged from 6.58 g to 13.59 g. Upon reaching the senescence stage, the weight of the leaves decreased, which was 5.70–10.38 g.

**Figure 1:**
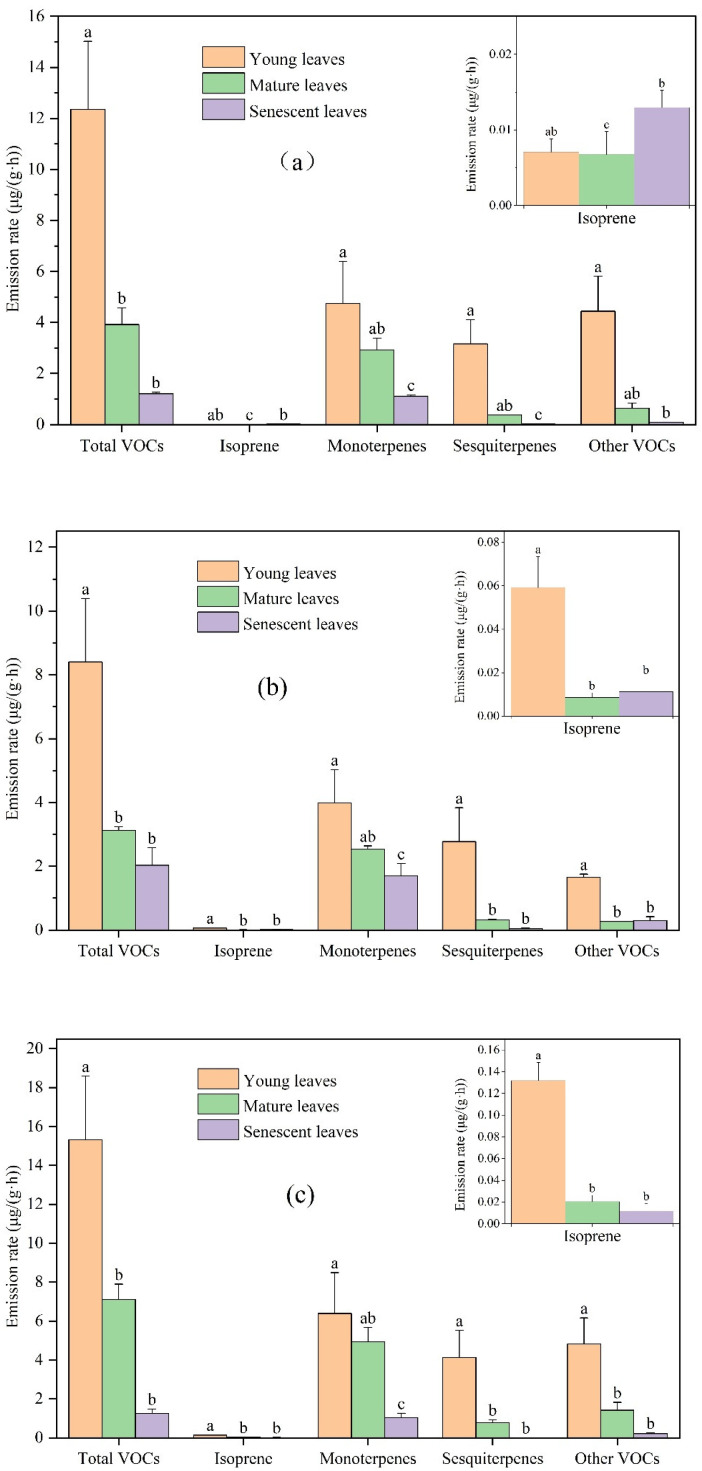
Differences in VOCs emission rates among young, mature, and senescent leaves for (**a**) *Ginkgo biloba*, (**b**) *Ligustrum lucidum*, and (**c**) *Forsythia suspensa*. Different letters in each picture indicate significant difference at 0.05 level.

There were significant leaf age-related differences in the emissions rate of total VOCs, isoprene, monoterpenes, sesquiterpenes, and other VOCs (Figure 1). VOCs emission rates of young leaves, mature leaves, and senescent leaves for *Ginkgo biloba* were 12.35, 3.92, and 1.21 μg/(g·h), respectively. VOCs emission rates of young leaves, mature leaves, and senescent leaves for *Ligustrum lucidum* were 8.40, 3.13, and 2.03 μg/(g·h), respectively. VOCs emission rates of young leaves, mature leaves, and senescent leaves for *Forsythia suspensa* were 15.32, 7.10, and 1.25 μg/(g·h), respectively. The main compounds emitted from the three plants at different leaf ages were monoterpenes, followed by other VOCs and sesquiterpenes, and isoprene emission was the least. Owen and Peñuelas suggested that the volatile terpenoid emissions might be controlled by the same factors that control the terpenoid pathway, producing the higher molecular weight, “essential” terpenoids [32,33]. These include, e.g., carotenoid pigments and gibberellins, the plant hormones regulating many developmental processes. Thus, the production of volatile terpenoids is high [34].

There are significant differences in the emission rates of *Ginkgo biloba*, *Ligustrum lucidum*, and *Forsythia suspensa* among different leaf ages, and most of the compounds had decreased emissions with increasing leaf age. For *Ginkgo biloba*, compared with young leaves, the emission rates of total VOCs, isoprene, monoterpenes, sesquiterpenes, and other VOCs in mature leaves decreased by 68.29%, 4.23%, 38.51%, 88.47%, and 85.79%, respectively. Compared with mature leaves, their emission rates in senescent leaves were changed by −69.23%, +91.10%, −62.11%, −96.63%, and −83.37%, respectively. For *Ligustrum lucidum*, compared with young leaves, the emission rates of total VOCs, isoprene, monoterpenes, sesquiterpenes, and other VOCs in mature leaves decreased by 62.79%, 85.55%, 36.22%, 88.41%, and 83.90%. Compared with mature leaves, the emission rates of total VOCs, isoprene, monoterpenes, sesquiterpenes, and other VOCs from senescent leaves were changed by −35.16%, +31.37%, −33.17%, −87.55%, and +9.20%, respectively. For *Forsythia suspensa*, compared with young leaves, the emission rates of total VOCs, isoprene, monoterpenes, sesquiterpenes, and other VOCs from mature leaves were reduced by 53.64%, 84.46%, 22.96%, 81.40%, and 70.63%, respectively. Compared with the mature leaves, the emission rates of total VOCs, isoprene, monoterpenes, sesquiterpenes, and other VOCs from senescent leaves decreased by 82.36%, 42.63%, 78.91%, 99.18%, and 85.25%, respectively.

Overall, the VOCs emission rate decreased with leaf age. The VOCs emission rate of all compounds from the young leaves of the studied plant species was higher than that from the mature leaves, and that from mature leaves was higher than that from senescent leaves for most compounds, which was consistent with previous studies. The release of VOCs is closely related to the enzyme activity. Research has shown that both VOCs emissions and enzyme activity increased by more than 100 times in the young leaves, while the enzyme activity decreased in the senescent leaves [35], which was consistent with the difference in the VOCs emission rate at different leaf ages in this study. In addition, the optimal defense hypothesis (ODH) states that organisms evolved to allocate their defenses in a way that maximizes fitness, in other words, the theoretical expectations of the ODH are that, within a plant, young, still-developing leaves should be better defended than older leaves [36]. Very young leaves usually still lack effective mechanical defenses, and the defense needs of young leaves are higher [36]. The cellular respiration of plants in the young leaves is higher than that in the mature and senescent leaves, while the metabolic activity of mature leaves is lower, resulting in a low rate of neogenesis [34]. Therefore, compared with mature and senescent leaves, young leaves of plants show a higher VOCs emission rate. VOCs are produced through the methylerythritol phosphate (MEP) pathway in chloroplasts [37]. During the aging period, the plant leaves gradually fade, the water in the leaves gradually decreases, and the photosynthesis and transpiration of the plant gradually weaken, which leads to the reduction of foliage VOCs emissions [22]. Study has shown that the stomata in senescent leaves remained closed even in the presence of light [38].

### 3.2. VOCs Emission Composition at Different Leaf Ages

**Figure 2:**
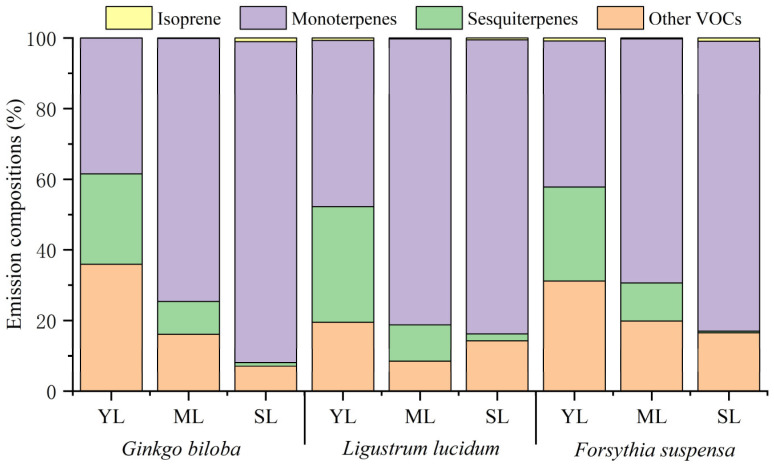
Differences in the VOCs emission composition among young leaves (YL), mature leaves (ML), and senescent leaves (SL) of *Ginkgo biloba*, *Ligustrum lucidum*, and *Forsythia suspensa*.

Leaf age effects on the emission proportions of isoprene, monoterpenes, sesquiterpenes, and other VOCs to the total VOCs are shown in Figure 2. With increasing leaf age, the proportion of isoprene for the three plant species showed different changes. Compared with young leaves, the proportion of isoprene emission by mature leaves of *Ginkgo biloba* increased by 0.12%, while that of *Ligustrum lucidum* and *Forsythia suspensa* decreased by 0.43% and 0.57%, respectively. Compared with mature leaves, the proportion of isoprene by senescent leaves increased by 0.28–0.90%. The proportion of monoterpenes in the total VOCs for the three plant species all showed an increasing trend. From young leaves to senescent leaves, the proportion increased by 53.38%, 36.33%, and 41.15% for *Ginkgo biloba*, *Ligustrum lucidum*, and *Forsythia suspensa*, respectively. Compared with young leaves, the proportion of monoterpenes by mature leaves increased by 27.61–36.12%. Compared with mature leaves, the proportion of monoterpenes by senescent leaves also increased by 2.49–17.26%. On the contrary, the proportion of sesquiterpenes emission decreased with increasing leaf age for all the three plant species. From young leaves to senescent leaves, the proportion of sesquiterpenes emission of *Ginkgo biloba* decreased by 24.60%, that of *Ligustrum lucidum* decreased by 31.01%, and that of *Forsythia suspensa* decreased by 26.38%. Compared with young leaves, the proportion of sesquiterpenes by mature leaves decreased by 16.10–22.71%, while that of monoterpenes by senescent leaves decreased by 8.29–10.28%. For other VOCs, compared with young leaves, the proportion of emission by mature leaves decreased by 11.13–19.82%. Compared with mature leaves, the proportion of emission by senescent leaves of *Ginkgo biloba* and *Forsythia suspensa* decreased by 8.97% and 3.26%, respectively, while that of *Ligustrum lucidum* increased by 5.81%. The contribution of each compound species to VOCs emissions changed significantly with leaf ages. The number of detected compounds at different stages were also different (Table S2), which was the highest for mature leaves, followed by young leaves and senescent leaves. The different behavior in plant species and the differences in their emission composition during development may reflect the functional diversity of these compounds at different developmental stages for plants.

The compounds with the highest emissions from the three plants at different leaf ages were all monoterpenes, but the specific dominated compounds were not consistent. The compound with the highest proportion of emission by young leaves of *Ginkgo biloba* and *Forsythia suspensa* was terpinolene, while it was α-terpiene for the mature and senescent leaves. The compounds with the highest proportion in different leaf ages for *Ligustrum lucidum* varied. Terpinolene had the highest proportion for young leaves, α-terpiene had the highest proportion for mature leaves, and α-pinene has the highest proportion for senescent leaves.

### 3.3. OFP and SOAP from VOC Emissions

#### 3.3.1. OFP and Its Composition

**Figure 3:**
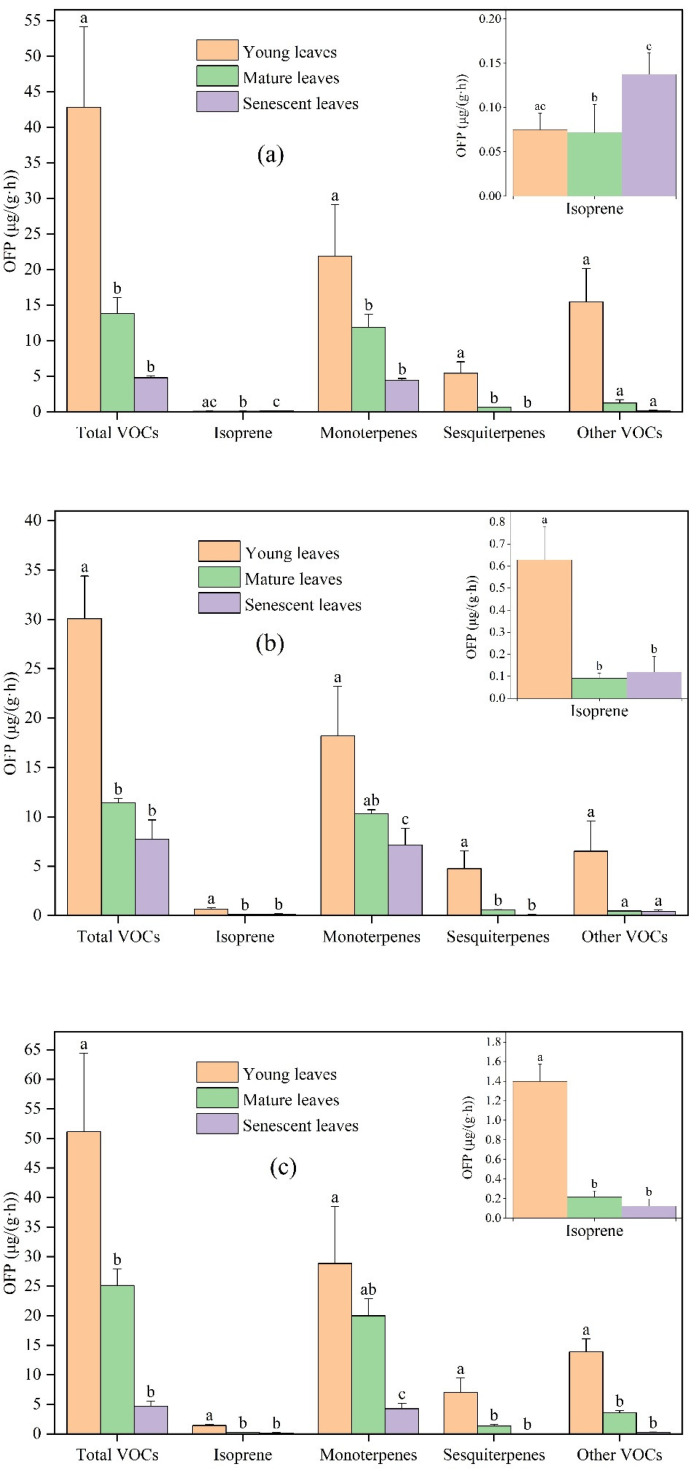
Differences in OFP from VOCs emissions among young, mature, and senescent leaves for (**a**) *Ginkgo biloba*, (**b**) *Ligustrum lucidum*, and (**c**) *Forsythia suspensa*. Different letters in each picture indicate significant difference at 0.05 level.

OFP and SOAP can reflect the reactive activity of various VOCs in the atmosphere and their relative contribution to O_3_ and SOA generation, which can help to identify the major VOCs active components [39]. The OFP and SOAP of total VOCs, isoprene, monoterpenes, sesquiterpenes, and other VOCs emissions were significantly different among different leaf ages (Figure 3).

In general, the OFP by young leaves was the highest, followed by mature leaves and senescent leaves. The OFP of *Ginkgo biloba* from young, mature, and senescent leaves was 42.83, 13.81, and 4.79 μg/(g·h), respectively. The OFP of *Ligustrum lucidum* from young, mature, and senescent leaves was 30.06, 11.42, and 7.75 μg/(g·h), respectively. The OFP of *Forsythia suspensa* from young, mature, and senescent leaves was 51.13, 25.11, and 4.64 μg/(g·h), respectively. The contribution of monoterpenes to total OFP was the highest, followed by sesquiterpenes and other VOCs, and the contribution of isoprene was the lowest.

For the studied plants, OFP was greatly different among different leaf ages. Compared with *Ginkgo biloba* young leaves, the OFP of total VOCs, isoprene, monoterpenes, sesquiterpenes, and other VOCs from mature leaves decreased by 67.76%, 4.23%, 45.68%, 88.47%, and 92.04%, respectively. Compared with *Ginkgo biloba* mature leaves, the OFP of total VOCs, isoprene, monoterpenes, sesquiterpenes, and other VOCs from senescent leaves changed by −65.28%, +91.10%, −62.51%, −96.63%, and −85.27%, respectively. Compared with *Ligustrum lucidum* young leaves, the OFP of total VOCs, isoprene, monoterpenes, sesquiterpenes and other VOCS from mature leaves decreased by 62.01%, 85.55%, 43.23%, 88.41%, and 93.03%, respectively. Compared with *Ligustrum lucidum* mature leaves, the OFP of total VOCs, isoprene, monoterpenes, sesquiterpenes, and other VOCs emissions from senescent leaves changed by −32.13%, +31.37%, −30.67%, −87.55%, and −10.83%, respectively. Compared with *Forsythia suspensa* young leaves, the OFP of total VOCs, isoprene, monoterpenes, sesquiterpenes, and other VOCS emissions from mature leaves decreased by 50.88%, 84.46%, 30.65%, 81.40%, and 74.06%, respectively. Compared with the *Forsythia suspensa* mature leaves, the OFP of total VOCs, isoprene, monoterpenes, sesquiterpenes, and other VOCs emissions from senescent leaves was reduced by 81.51%, 42.63%, 78.76%, 99.18%, and 92.74%, respectively. It is worth noting that OFP is estimated based on VOC emissions. For most compounds, the difference in OFP and VOCs emission at different leaf ages was similar, but there were also some exceptions. For example, compared to mature leaves, the emission rate of other VOCs by senescent leaves of *Ligustrum lucidum* increased by 9.20%, while the OFP decreased by 10.83%. Although the emission rate of other VOCs by mature leaves was lower than that of senescent leaves, alkenes accounted for a higher proportion of other VOCs emission by mature leaves, and alkenes had high chemical reaction activity; the MIR of cis-2-pentene and trans-2-pentene was as high as 10.38 and 10.56 g O_3_/g VOCs, respectively.

**Figure 4:**
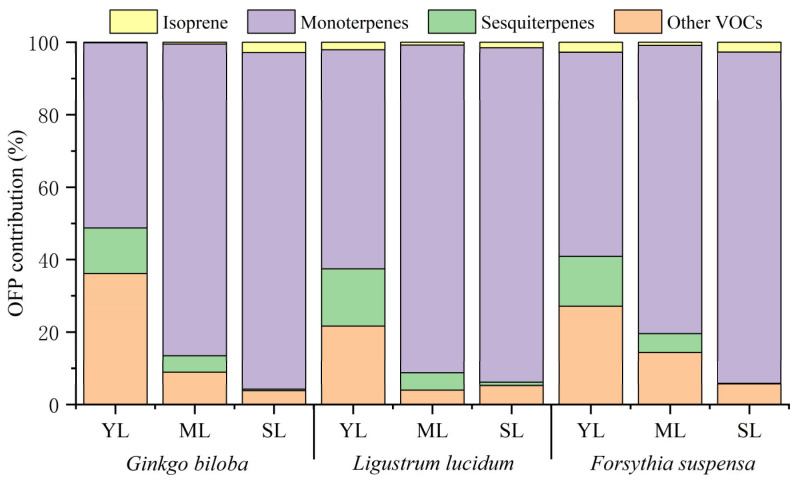
Differences in the OFP contribution by VOCs categories among young leaves (YL), mature leaves (ML), and senescent leaves (SL) of *Ginkgo biloba*, *Ligustrum lucidum*, and *Forsythia suspensa*.

The contribution of isoprene, monoterpenes, sesquiterpenes, and other VOCs to the total OFP varied among different leaf ages (Figure 4). With increasing leaf age, the contribution of isoprene of the three plant species showed different changes. Compared with young leaves, the OFP contribution of isoprene from mature leaves of *Ginkgo biloba* increased by 0.35%, while that of *Ligustrum lucidum* and *Forsythia suspensa* decreased by 1.30% and 1.87%. Compared with mature leaves, the OFP contribution of isoprene from senescent leaves increased by 0.74–2.35%. The OFP contribution of monoterpenes for the three plant species all showed an increasing trend. Compared with young leaves, the OFP contribution of monoterpenes from mature leaves increased significantly, by 23.23–34.97%. Compared with mature leaves, the OFP contribution of monoterpenes from senescent leaves increased slightly, by 1.94–11.86%. On the contrary, the OFP contribution of sesquiterpenes decreased with increasing leaf age for all three species. Compared with young leaves, the OFP contribution of sesquiterpenes from mature leaves decreased by 8.12–10.96%. Compared with mature leaves, the OFP contribution of sesquiterpenes from senescent leaves decreased by 3.93–4.98%. For other VOCs, compared with young leaves, the OFP contribution from mature leaves decreased by 12.80–27.20%; compared with mature leaves, the OFP contribution from senescent leaves of *Ginkgo biloba* and *Forsythia suspensa* decreased by 5.14% and 8.70%, respectively, while that of *Ligustrum lucidum* increased by 1.25%.

#### 3.3.2. SOAP and Its Composition

**Figure 5:**
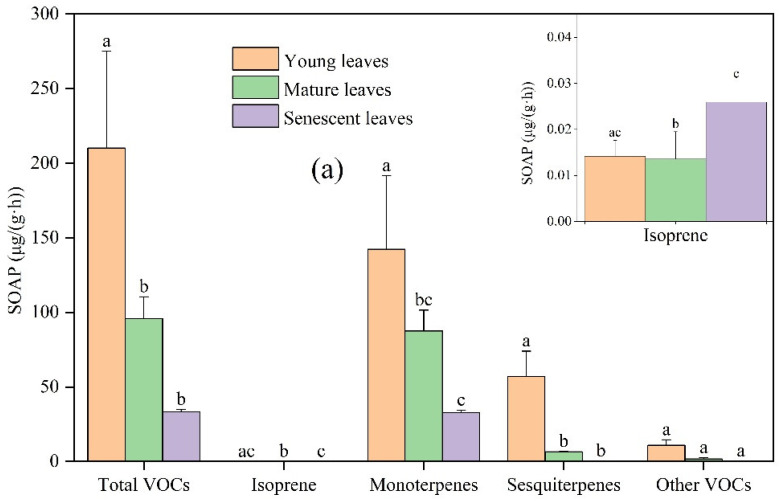

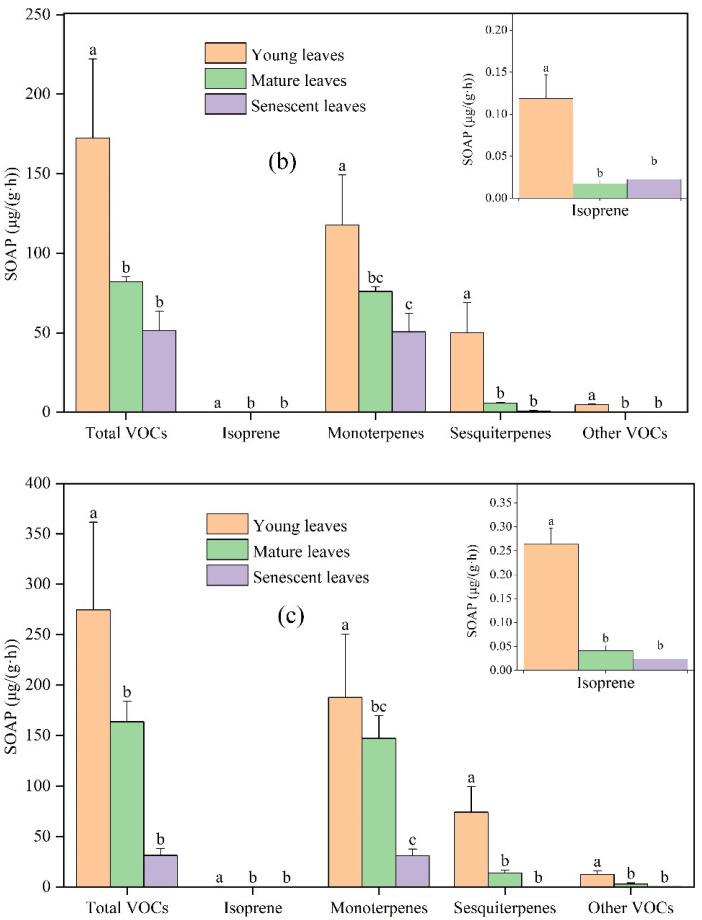
Differences in SOAP from VOCs emissions among young, mature, and senescent leaves for (**a**) *Ginkgo biloba*, (**b**) *Ligustrum lucidum*, and (**c**) *Forsythia suspensa*. Different letters in each picture indicate significant difference at 0.05 level.

Overall, the total SOAP was the highest in young leaves, followed by mature leaves and the lowest in senescent leaves (Figure 5). The SOAP from young leaves of *Ginkgo biloba*, mature leaves, and senescent leaves was 210.14, 95.70, and 33.27 μg/(g·h), respectively. The SOAP from young, mature, and senescent leaves of *Ligustrum lucidum* was 172.46, 81.88, and 51.44 μg/(g·h), respectively. The SOAP from young, mature, and senescent leaves of *Forsythia suspensa* was 274.55, 163.59, and 31.24 μg/(g·h), respectively. Monoterpenes contributed the highest SOAP, followed by sesquiterpenes and other VOCs, and isoprene contributed the lowest to the total SOAP.

With increasing leaf age, the SOAP values of *Ginkgo biloba*, *Ligustrum lucidum*, and *Forsythia suspensa* were greatly different. For *Ginkgo biloba*, compared with young leaves, the SOAP of total VOCs, isoprene, monoterpenes, sesquiterpenes, and other VOCs from mature leaves decreased by 54.46%, 4.23%, 38.56%, 88.47%, and 84.49%, respectively; compared with mature leaves, the SOAP of total VOCs, isoprene, monoterpenes, sesquiterpenes, and other VOCs from senescent leaves changed by −65.24%, +91.10%, −62.47%, −96.63%, and −87.77%, respectively. For *Ligustrum lucidum*, compared with young leaves, the SOAP of total VOCs, isoprene, monoterpenes, sesquiterpenes, and other VOCs from mature leaves decreased by 52.52%, 85.55%, 35.48%, 88.41%, and 97.54%, respectively; compared with mature leaves, the SOAP of total VOCs, isoprene, monoterpenes, sesquiterpenes, and other VOCs from senescent leaves changed by −37.18%, +31.37%, −33.39%, −87.55%, and −15.78%, respectively. Compared with *Forsythia suspensa* young leaves, the SOAP of total VOCs, isoprene, monoterpenes, sesquiterpenes, and other VOCs from mature leaves decreased by 40.41%, 84.46%, 21.67%, 81.40%, and 78.35%, respectively. Compared with the *Forsythia suspensa* mature leaves, the SOAP of total VOCs, isoprene, monoterpenes, sesquiterpenes, and other VOCS from senescent leaves decreased by 80.90%, 42.63%, 79.06%, 99.18%, and 88.56%, respectively.

**Figure 6:**
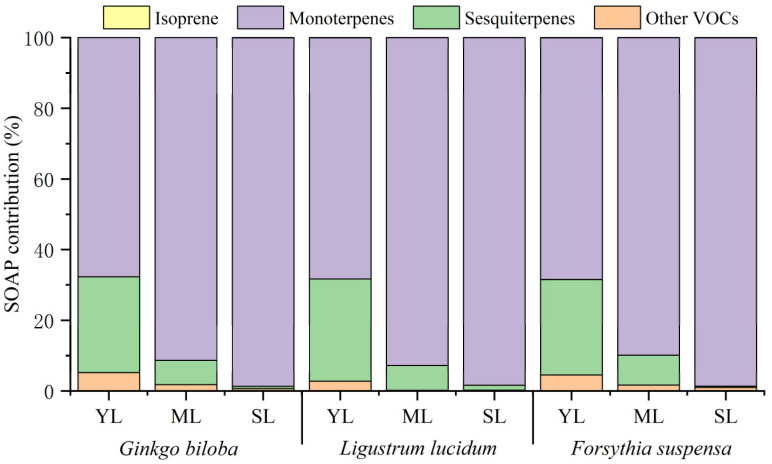
Differences in the SOAP contribution by VOCs categories among young leaves (YL), mature leaves (ML), and senescent leaves (SL) of *Ginkgo biloba*, *Ligustrum lucidum*, and *Forsythia suspensa*.

The contribution of isoprene, monoterpenes, sesquiterpenes, and other VOCs to the total SOAP was different among different leaf ages (Figure 6). With increasing leaf age, the proportion of isoprene contribution for the three plant species showed different changes. Compared with young leaves, the SOAP contribution of isoprene by mature leaves of *Ginkgo biloba* increased by 0.01%, while the SOAP contribution of *Ligustrum lucidum* and *Forsythia suspensa* decreased by 0.05% and 0.07%, respectively. Compared with mature leaves, the SOAP contribution of isoprene from senescent leaves increased by 0.02–0.06%. The SOAP contribution of monoterpenes for the three plant species all showed an increasing trend. Compared with young leaves, the SOAP contribution of monoterpenes from mature leaves increased significantly, by 21.52–24.51%. Compared with mature leaves, the SOAP contribution of monoterpenes from senescent leaves increased slightly, by 5.59–8.67%. On the contrary, the SOAP contribution of sesquiterpenes decreased with leaf age for all three plant species. Compared with young leaves, the SOAP contribution of sesquiterpenes from mature leaves decreased by 18.57–21.87%. Compared with mature leaves, the SOAP contribution of sesquiterpenes from senescent leaves decreased by 5.66–8.07%. For other VOCs, compared with young leaves, the SOAP contribution for emission by mature leaves decreased by 2.59–3.40%. Compared with mature leaves, the SOAP contribution of other VOCs emission from senescent leaves of *Ginkgo biloba* and *Forsythia suspensa* decreased by 1.14% and 0.66%, respectively, while that of *Ligustrum lucidum* increased by 0.05%.

In summary, monoterpenes contributed the most to the formation of O_3_ and SOA, while isoprene contributed the least. The proportions of OFP and SOAP by other VOCs and sesquiterpenes in the total varied in magnitude. It is important to note that high VOCs emissions may not result in high OFP and SOAP. In addition to the VOCs emission level, the composition of VOCs also affects their OFP and SOAP. Different compounds have different chemical structures and chemical reaction activities, resulting in different MIR values and FAC coefficients, which lead to different OFP and SOAP [40]. For example, the VOCs emission rates of isoprene and sesquiterpene for senescent leaves of *Ginkgo biloba* were near, 0.012 and 0.013 μg/(g·h), respectively, while a difference by one order of magnitude existed in their OFP and SOAP, with the OFP of 0.14 and 0.02 μg/(g·h) and SOAP of 0.03 and 0.22 μg/(g·h), respectively. This is because the MIR value of isoprene is high, 10.61 g O_3_/g VOCs, and that of sesquiterpenes is only 1.71 g O_3_/g VOCs, while the FAC value of sesquiterpenes is much higher, 18%, and that of isoprene is only 2%. This results in a significant difference in the identified dominated VOCs species based on emissions and potentials to form secondary air pollutants. It should be referred to in the control of complex air pollution.

## 4. Conclusions

In this study, VOCs emissions of *Ginkgo biloba*, *Ligustrum lucidum*, and *Forsythia suspensa* at different leaf ages (young, mature, and senescent leaves) were collected and analyzed using a dynamic enclosure system and TD–GC–MS technique. Their OFP and SOAP were estimated based on the emission of quantified 63 VOCs. Plants at different leaf ages had had significantly different VOCs emission intensity and composition. VOCs emission was the highest in young leaves and the lowest in senescent leaves. Monoterpenes and isoprene were, respectively, the highest and lowest contributors to total VOCs emissions. With increasing leaf age, the emission contribution of monoterpenes increased, while sesquiterpenes decreased. The total OFP and SOAP and their composition by isoprene, monoterpenes, sesquiterpenes, and other VOCs also varied significantly among different leaf ages. VOCs emission from young leaves had the highest OFP and SOAP, while they were the lowest from senescent leaves. With increasing leaf age, the contribution of monoterpenes to OFP and SOAP increased, and that of sesquiterpenes decreased. For leaves at different ages, the VOCs composition by contributing to the emission, OFP, and SOAP varied. When studying the precise prevention and control of secondary air pollution and selecting greening plants to mitigate VOCs emissions, it should be considered comprehensively.

## Data Availability

The data presented in this study are available on request from the corresponding author.

## Supplementary Materials

The following supporting information can be downloaded at: https://www.mdpi.com/article/10.3390/toxics11080645/s1, Text S1: Emission rate standardization; Table S1: Profile of pure standard samples, detection limits, analysis precision, MIR and FAC values for each compound; Table S2: Compound-specific emission rates from the young, mature, and senescent leaves of *Ginkgo biloba*, *Ligustrum lucidum*, and *Forsythia suspense* (Units: μg/(g·h); ND: not detected); Table S3: Changes of VOCs emission rates between leaves of different ages at *Ginkgo biloba*, *Ligustrum lucidum*, and *Forsythia suspense* (Compared with the previous growth stage) (Units: %); Table S4: Changes of VOCs emission composition between leaves of different ages at *Ginkgo biloba*, *Ligustrum lucidum*, and *Forsythia suspense* (Compared with the previous growth stage) (Units: %); Table S5: Changes of OFP between leaves of different ages at *Ginkgo biloba*, *Ligustrum lucidum*, and *Forsythia suspense* (Compared with the previous growth stage) (Units: %); Table S6: Changes of OFP contribution between leaves of different ages at *Ginkgo biloba*, *Ligustrum lucidum*, and *Forsythia suspense* (Compared with the previous growth stage) (Units: %); Table S7: Changes of SOAP between leaves of different ages at *Ginkgo biloba*, *Ligustrum lucidum*, and *Forsythia suspense* (Compared with the previous growth stage) (Units: %); Table S8: Changes of SOAP contribution between leaves of different ages at *Ginkgo biloba*, *Ligustrum lucidum*, and *Forsythia suspense* (Compared with the previous growth stage) (Units: %).
