# Supplementary material for: Variations in VOCs Emissions and Their O3 and SOA Formation Potential among Different Ages of Plant Foliage
Source: Toxics. 2023 Jul 25;11(8):645. doi: 10.3390/toxics11080645 (PMC10458546; doi:10.3390/toxics11080645)
Supplement: Supplementary file 1 [file toxics-11-00645-s001.zip › toxics-2490218-supplementary.pdf]

toxics

Supplementary Materials

Variations in VOCs Emissions and Their O_3 and SOA Formation Potential among Different Ages of Plant Foliage

Baowen Zhang, Lili Qiao, Huijuan Han, Wenxia Xie * and Lingyu Li *

College of Environmental Sciences and Engineering, Carbon Neutrality and Eco-Environmental Technology Innovation Center of Qingdao, Qingdao University, Qingdao 266071, China; zhangbaowen28@163.com (B.Z.); 13210158173@163.com (L.Q.); hanhuijuan2022@163.com (H.H.)

* Correspondence: xwx080312@163.com (W.X.); lilingyu@qdu.edu.cn (L.L.)

Citation: Zhang, B.; Qiao, L.; Han, H.; Xie, W.; Li, L. Variations in VOCs Emissions and Their O_3 and SOA Formation Potential among Different Ages of Plant Foliage. *Toxics* **2023**, *11*, 645. https://doi.org/10.3390/toxics11080645

Academic Editor: Yuan Cheng

Received: 21 June 2023
Revised: 21 July 2023
Accepted: 22 July 2023
Published: 25 July 2023

Copyright: © 2023 by the authors. Licensee MDPI, Basel, Switzerland. This article is an open access article distributed under the terms and conditions of the Creative Commons Attribution (CC BY) license (https://creativecommons.org/licenses/by/4.0/).

Text S1. Emission rate standardization.

Referring to Guenther et al. [28], the actual emission rates of each VOC's component were converted to the standard ones under the standard conditions of temperature=30°C and PAR=1000 μmol/(m^2·s) using Equation (S1).

$$EF_{s,i}=EF_i\times \gamma_{P,i}\times\gamma_{T,i} \tag{S1}$$

where $EF_{s,i}$ is the emission rate at standard conditions; μg/(g·h); EF_i is the emission rate under the real environment of VOC_i; and μg/(g·h), $\gamma_{P,i}$, and $\gamma_{T,i}$ are dimensionless activity factors which correct for light and temperature conditions, respectively.

The PAR correction factor $\gamma_{P,i}$ is calculated as follows.

$$\gamma_{P,i}=(1-LDF_i)+LDF_i\times C_L \tag{S2}$$

where LDF_i is the light-dependent fraction; it is 1 for isoprene; 0.6 for myrcene, sabinene, and α-pinene; 0.2 for limonene, 3-carene, and β-pinene; 0.8 for trans-β-ocimene; 0.4 for other monoterpenes; 0.5 for sesquiterpenes; and 0.2 for other VOCs. C_L is the light-dependent activity factor, calculated as Equation (S3).

$$C_L=\frac{\alpha C_{L1}L}{\sqrt{1+\alpha^2L^2}} \tag{S3}$$

where L is the PAR, μmol/(m^2·s); α is 0.0027; and C_{L1} is the empirical constant, 1.006.

The temperature-correction factor $\gamma_{T,i}$ is calculated as follows.

$$\gamma_{T,i}=(1-LDF_i)\gamma+LDF_i\times C_T \tag{S4}$$

where LDF_i is the light-dependent fraction; γ is calculated as Equation (S6); and C_T is the temperature-dependent activity factor, calculated as follows.

$$C_T=\frac{\exp[\frac{C_{T1}(T-T_S)}{RT_ST}]}{\{1+\exp\left[\frac{C_{T2}(T-T_M)}{RT_ST}\right]\}} \tag{S5}$$

where T is the leaf temperature, K; T_S is the leaf temperature at standard conditions, and it is 303 K; R is 8.314 J/(K·mol); C_{T1} is the empirically determined coefficient, and it is 95000 J/mol for isoprene, 80000 J/mol for monoterpene, 130000 J/mol for sesquiterpene, and 80000 J/mol for other VOCs; C_{T2} is 230000 J/mol; and T_M is 314 K.

$$\gamma=\exp[\beta(T-T_S)] \tag{S6}$$

where β is the empirical constant, and it is 0.13 for isoprene, 0.1 for monoterpene, 0.17 for sesquiterpene, and 0.1 for other VOCs; T is the leaf temperature, K; and T_S is the leaf temperature at standard conditions, 303 K.

Table S1. Profile of pure standard samples, detection limits, analysis precision, and MIR and FAC values for each compound.

Compound	Classification	Detection limits (μg/m³)	Precision (%)	MIR (g O_3/g VOCs)	FAC (%)
Isoprene	Isoprene	0.21	4	10.61	2
Tricyclene	Monoterpene	0.03	4	4.04	30
α-Pinene	Monoterpene	0.42	9	4.51	30
Camphene	Monoterpene	0.18	5	4.51	30
Sabinene	Monoterpene	0.99	10	4.19	30
β-Pinene	Monoterpene	0.68	8	3.52	30
Myrcene	Monoterpene	0.18	4	4.04	30
α-Phellandrene	Monoterpene	0.24	5	4.04	30
3-Carene	Monoterpene	0.25	4	3.24	30
α-Terpiene	Monoterpene	0.18	4	4.04	30
Limonene	Monoterpene	0.2	4	4.55	30
cis-β-Ocimene	Monoterpene	0.16	5	4.04	30
trans-β-Ocimene	Monoterpene	0.05	4	4.04	30
γ-Terpinene	Monoterpene	0.17	4	4.04	30
Terpinolene	Monoterpene	0.19	4	6.36	30
Isolongifolene	Sesquiterpene	0.07	5	1.71	18
Longifolene	Sesquiterpene	0.05	5	1.71	18
α-Cedrene	Sesquiterpene	0.07	5	1.71	18
β-Caryophyllene	Sesquiterpene	0.05	4	1.71	18
Aromadendrene	Sesquiterpene	0.15	7	1.71	18
α-Farnesene	Sesquiterpene	0.09	3	1.71	18
Cyclopentane	Alkane	0.18	10	2.39	4
2,2-Dimethylbutane	Alkane	0.14	8	1.17	0
2,3-Dimethylbutane	Alkane	0.39	5	0.97	0
2,4-Dimethylpentane	Alkane	0.52	7	1.55	0.06
2,3-Dimethylpentane	Alkane	0.31	8	1.34	0.06
3-Methylhexane	Alkane	0.29	7	1.61	0.06
n-Heptane	Alkane	0.28	10	1.07	0.06
2,3,4-Trimethylpentane	Alkane	0.44	15	1.03	0.73
2-Methylheptane	Alkane	0.37	10	1.07	0.5
3-Methylheptane	Alkane	0.42	10	1.24	0.5
n-Octane	Alkane	0.48	8	0.9	0.06
Methylcyclopentane	Alkane	0.43	8	2.19	0.17
3-Methylpentane	Alkane	0.42	13	1.8	0.17
n-Hexane	Alkane	0.2	9	1.24	0
2,2,4-Trimethylpentane	Alkane	0.19	9	1.26	0.73
n-Nonane	Alkane	0.26	13	0.78	1.5
n-Decane	Alkane	0.25	14	0.68	2
n-Undecane	Alkane	0.85	8	0.61	2.5
Dodecane	Alkane	0.31	10	0.55	3
Methylcyclohexane	Alkane	0.4	8	1.7	2.7
Cyclohexane	Alkane	0.41	6	1.25	0.17
1-Hexene	Alkene	0.34	3	5.49	-
1-Pentene	Alkene	0.88	7	7.21	-
trans-2-Pentene	Alkene	0.26	5	10.56	-
cis-2-Pentene	Alkene	0.33	15	10.38	-
Benzene	Aromatic	0.33	14	0.72	2

Toluene	Aromatic	0.46	10	4	5.4
Ethylbenzene	Aromatic	0.35	9	3.04	5.4
m-Xylene	Aromatic	0.72	14	9.75	4.7
p-Xylene	Aromatic	0.72	14	5.84	1.6
o-Xylene	Aromatic	0.35	9	7.64	5
n-Propylbenzene	Aromatic	0.27	5	2.03	1.6
Styrene	Aromatic	0.25	5	1.73	5.7
iso-Propylbenzene	Aromatic	0.32	7	2.52	4
m-Ethyltoluene	Aromatic	0.36	5	7.39	6.3
p-Ethyltoluene	Aromatic	0.33	13	4.44	2.5
1,3,5-Trimethylbenzene	Aromatic	0.36	13	11.76	2.9
o-Ethyltoluene	Aromatic	0.27	5	5.59	5.6
1,2,4- Trimethylbenzene	Aromatic	0.25	4	8.87	2
1,2,3- Trimethylbenzene	Aromatic	0.25	4	11.97	3.6
m-Diethylbenzene	Aromatic	0.23	4	7.1	6.3
p-Diethylbenzene	Aromatic	0.2	4	4.43	6.3

Note: MIR is the maximum incremental reactivity of VOCs. FAC is the aerosol formation coefficient of the VOCs. MIR values are from study [29], FAC values are from studies [30,31].

Table S2. Compound-specific emission rates from the young, mature, and senescent leaves of *Ginkgo biloba*, *Ligustrum lucidum*, and *Forsythia suspense* (Units: μg/(g·h); ND: not detected).

**Emission rate**	***Ginkgo biloba***			***Ligustrum lucidum***			***Forsythia suspensa***		
	**Young leaves**	**Mature leaves**	**Senescent leaves**	**Young leaves**	**Mature leaves**	**Senescent leaves**	**Young leaves**	**Mature leaves**	**Senescent leaves**
Isoprene	0.0071±0.0017	0.0068±0.003	0.0130±0.0023	0.0592±0.0142	0.0086±0.0022	0.0113±0.0026	0.1319±0.0167	0.0205±0.0058	0.0118±0.0023
Tricyclene	0.2768±0.1190	0.0004±0.0001	0.0004±0.0001	0.2343±0.0812	0.001±0.0004	0.0022±0.0015	0.3723±0.1353	0.0006±0.0001	0.0007±0.0002
α-Pinene	0.0010±0.0001	0.2506±0.0024	0.1019±0.0222	0.0261±0.0140	0.2175±0.0080	0.7232±0.1907	0.0032±0.0004	0.422±0.004	0.2645±0.0902
Camphene	0.0005±0.0002	0.0017±0.0005	0.0043±0.0005	0.0006±0.0002	0.0048±0.0018	0.0251±0.0111	0.004±0.0012	0.0033±0.0006	0.0104±0.0034
Sabinene	0.1230±0.0545	0.4741±0.0001	ND	0.0775±0.0001	0.4094±0.0167	0.2201±0.0001	0.1652±0.0554	0.7911±0.0401	ND
β-Pinene	0.0012±0.0001	0.4297±0.0033	0.0996±0.0009	0.0004±0.0003	0.3707±0.0139	0.1749±0.0336	0.0049±0.0027	0.7229±0.0083	0.0991±0.0248
Myrcene	0.7300±0.3138	0.3788±0.0039	0.1462±0.0011	0.6249±0.2258	0.3267±0.0124	0.0861±0.0090	0.9673±0.3202	0.6370±0.0068	0.1139±0.0079
α-Phellandrene	0.0014±0.0010	0.2697±0.0029	0.0384±0.0093	0.0002±0.0001	0.2333±0.0084	0.0247±0.0086	0.0051±0.0010	0.4545±0.0049	0.0172±0.0034
3-Carene	0.0005±0.0004	0.0002±0.0001	0.0032±0.0007	0.0015±0.0015	0.0052±0.0031	0.0162±0.0074	0.0030±0.0017	0.0003±0.0001	0.0025±0.0006
α-Terpiene	0.8603±0.3591	0.5262±0.0054	0.5699±0.0047	0.7310±0.2672	0.4521±0.0174	0.3171±0.0109	1.1131±0.3712	0.8862±0.0101	0.4354±0.0264
Limonene	0.0025±0.0001	0.2715±0.0043	0.0632±0.0105	ND	0.2393±0.0095	0.0410±0.0199	0.0094±0.0032	0.4592±0.0042	0.0283±0.0018
cis-β-Ocimene	0.4494±0.1970	0.1879±0.0027	ND	0.3715±0.1193	0.1636±0.0057	ND	0.6111±0.2004	0.3173±0.0032	ND
trans-β-Ocimene	0.3054±0.1283	0.1229±0.0011	0.0626±0.0004	0.2617±0.0951	0.1076±0.0042	0.0361±0.0019	0.4060±0.1336	0.2065±0.0023	0.0488±0.0032
γ-Terpinene	0.8327±0.2138	ND	0.0009±0.0003	0.6008±0.0001	ND	0.0024±0.0008	1.0825±0.3689	ND	0.0019±0.0005

Terpinolene	1.1592±0.2910	0.0008±0.0003	0.0032±0.0012	0.9940±0.3638	0.0010±0.0005	0.0176±0.0070	1.5118±0.5045	0.002±0.0006	0.0040±0.0006
Isolongifolene	0.4333±0.2164	0.0021±0.0002	ND	0.3859±0.1420	0.0004±0.0001	ND	0.5759±0.2035	0.0007±0.0001	ND
Longifolene	0.3241±0.1604	ND	ND	0.2890±0.1047	ND	0.0350±0.0159	0.4357±0.1491	0.1648±0.0001	ND
α-Cedrene	0.3846±0.1921	0.0503±0.0009	ND	0.3426±0.1258	0.0436±0.0029	ND	0.5114±0.1811	0.0833±0.0015	ND
β-Caryophyllene	0.5561±0.1328	0.0973±0.0022	ND	0.6060±0.2106	0.0894±0.0103	ND	0.9609±0.2885	0.1591±0.0049	ND
Aromadendrene	0.7840±0.0745	0.1278±0.0029	0.0123±0.0018	0.5049±0.2111	0.1131±0.0077	0.0050±0.0007	0.7104±0.2516	0.2173±0.0047	0.0063±0.0008
α-Farnesene	0.6824±0.3692	0.0873±0.0032	ND	0.6435±0.2685	0.0748±0.0046	ND	0.9238±0.3307	0.1406±0.0025	ND
Cyclopentane	ND	0.0048±0.0012	0.0042±0.0009	ND	0.0212±0.0027	ND	ND	0.0326±0.0082	ND
2,2-Dimethylbutane	ND	ND	0.0512±0.0081	ND	0.0118±0.0066	0.0159±0.0061	ND	ND	0.0361±0.0044
2,3-Dimethylbutane	ND	0.0639±0.0001	ND	ND	0.0514±0.0447	0.0003±0.0001	ND	0.0555±0.0028	ND
2,4-Dimethylpentane	ND	0.0587±0.0068	0.0248±0.0081	ND	0.0106±0.0028	0.0284±0.0106	ND	0.1474±0.0062	0.0519±0.0083
2,3-Dimethylpentane	ND	ND	ND	ND	0.0183±0.0075	0.0606±0.0261	ND	ND	0.0094±0.0019
3-Methylhexane	ND	ND	ND	ND	ND	0.1217±0.0001	ND	ND	ND
n-Heptane	ND	ND	ND	ND	ND	ND	ND	0.1852±0.0115	ND
2,3,4-Trimethylpentane	ND	ND	ND	ND	ND	ND	ND	ND	ND
2-Methylheptane	ND	ND	ND	ND	ND	ND	0.0701±0.0001	ND	ND
3-Methylheptane	ND	0.0664±0.0135	ND	ND	ND	0.0068±0.0004	ND	0.1095±0.0131	ND

n-Octane	0.0712±0.0155	ND	ND	ND	0.0872±0.0094	ND	0.0426±0.0001	ND	ND
Methylcyclopentane	0.1278±0.0585	0.0190±0.0001	ND	ND	0.0077±0.0059	0.0119±0.0001	0.1636±0.0474	0.0297±0.0057	0.0029±0.0007
3-Methylpentane	0.0318±0.0001	ND	0.0052±0.0013	ND	0.0018±0.0004	0.0079±0.0022	ND	0.0051±0.0017	0.0199±0.0036
n-Hexane	ND	0.0230±0.0117	ND	ND	0.0442±0.0134	ND	ND	0.0807±0.0188	ND
2,2,4-Trimethylpentane	ND	0.0056±0.0001	ND	0.0115±0.0001	0.0032±0.0013	0.0254±0.0017	ND	ND	ND
n-Nonane	ND	0.0026±0.0019	ND	ND	0.0005±0.0002	ND	ND	ND	ND
n-Decane	0.3090±0.0639	0.0055±0.0020	ND	ND	ND	ND	0.2587±0.0001	0.0077±0.0012	0.0325±0.0001
n-Undecane	0.4382±0.0638	0.0079±0.0025	ND	ND	ND	ND	0.5608±0.1916	0.0127±0.0005	0.0546±0.0001
Dodecane	0.9658±0.0001	ND	ND	0.7227±0.3162	ND	ND	0.8322±0.2589	0.0032±0.0001	ND
Methylcyclohexane	ND	0.0095±0.0065	ND	ND	ND	ND	1.3156±0.3891	ND	ND
Cyclohexane	ND	ND	ND	ND	ND	ND	ND	ND	ND
1-Hexene	ND	ND	0.0006±0.0002	ND	ND	0.0019±0.0005	ND	0.0135±0.0001	0.0010±0.0003
1-Pentene	ND	0.0048±0.0012	0.0042±0.0009	ND	0.0212±0.0027	ND	ND	0.0326±0.0082	ND
trans-2-Pentene	0.6930±0.0001	ND	ND	ND	ND	ND	ND	0.1572±0.0001	ND
cis-2-Pentene	ND	ND	ND	ND	ND	ND	ND	ND	ND
Benzene	ND	ND	ND	ND	ND	ND	ND	ND	ND
Toluene	ND	0.0185±0.0032	ND	ND	0.0075±0	0.0056±0.0012	ND	0.0141±0.0005	ND
Ethylbenzene	0.1117±0.0012	0.0154±0.0032	ND	0.0647±0.0001	0±0	0±0	0.1309±0.0491	0.0357±0.0009	ND

m-Xylene	0.2482±0.0001	0.0038±0.0009	ND	0.1824±0.0001	0±0	0±0	ND	0.0088±0.0010	ND
p-Xylene	0.2490±0.0001	0.0100±0.0071	ND	ND	ND	ND	ND	0.0121±0.0015	ND
o-Xylene	0.3082±0.0001	0.0100±0.0071	ND	ND	ND	ND	ND	0.0121±0.0015	ND
n-Propylbenzene	0.1405±0.0001	0.0048±0.0039	ND	ND	ND	ND	0.2483±0.0001	0.0053±0.0017	ND
Styrene	0.2603±0.0455	0.0492±0.0005	ND	ND	ND	0.0037±0.0005	ND	0.0827±0.0008	ND
iso-Propylbenzene	ND	0.1579±0.0112	ND	ND	ND	ND	ND	0.2456±0.0001	ND
m-Ethyltoluene	0.3124±0.0001	0.0884±0.0003	ND	ND	ND	ND	ND	0.1468±0.0001	ND
p-Ethyltoluene	0.1701±0.0001	0.0042±0.0024	ND	0.0009±0.0001	ND	ND	0.0142±0.0001	0.0097±0.0001	ND
1,3,5-Trimethylbenzene	ND	0.0007±0.0004	ND	0.2395±0.0247	0.0003±0.0001	ND	0.3983±0.1421	0.0004±0.0001	ND
o-Ethyltoluene	ND	0.0009±0.0003	ND	0.1285±0.0114	ND	ND	0.2069±0.0662	ND	ND
1,2,4-Trimethylbenzene	ND	ND	ND	ND	ND	0.0001±0.0001	ND	ND	ND
1,2,3-Trimethylbenzene	ND	ND	ND	0.2992±0.0264	ND	ND	0.5698±0.1982	ND	ND
m-Diethylbenzene	ND	ND	ND	ND	ND	ND	ND	ND	ND
p-Diethylbenzene	ND	ND	ND	ND	ND	ND	ND	ND	ND

Table S3. Changes in VOC emission rates between leaves of different ages for *Ginkgo biloba*, *Ligustrum lucidum*, and *Forsythia suspensa* (Compared with the previous growth stage) (Units: %).

Plant	Compound	Young leaves	Mature leaves	Senescent leaves
Ginkgo biloba	Total VOCs	—	−68.29	−69.23
	Isoprene	—	−4.23	91.10
	Monoterpenes	—	-38.51	−62.11
	Sesquiterpenes	—	−88.47	−96.63
	Other VOCs	—	−85.79	−83.37
Ligustrum lucidum	Total VOCs	—	−62.79	−35.16
	Isoprene	—	−85.55	31.37
	Monoterpenes	—	−36.22	−33.17
	Sesquiterpenes	—	−88.41	−87.55
	Other VOCs	—	−83.90	9.20
Forsythia suspensa	Total VOCs	—	−53.64	−82.36
	Isoprene	—	−84.46	−42.63
	Monoterpenes	—	−22.96	−78.91
	Sesquiterpenes	—	−81.40	−99.18
	Other VOCs	—	−70.63	−85.25

Table S4. Changes in VOCs′ emission composition between leaves of different ages for *Ginkgo biloba*, *Ligustrum lucidum*, and *Forsythia suspensa* (Compared with the previous growth stage) (Units: %).

Plant	Compound	Young leaves	Mature leaves	Senescent leaves
Ginkgo biloba	Isoprene	—	0.12	0.90
	Monoterpenes	—	36.12	17.26
	Sesquiterpenes	—	−16.30	−8.29
	Other VOCs	—	−19.82	−8.97
Ligustrum lucidum	Isoprene	—	−0.43	0.28
	Monoterpenes	—	33.84	2.49
	Sesquiterpenes	—	−22.71	−8.30
	Other VOCs	—	−11.13	5.81
Forsythia suspensa	Isoprene	—	−0.57	0.65
	Monoterpenes	—	27.61	13.55
	Sesquiterpenes	—	−16.10	−10.28
	Other VOCs	—	−11.51	−3.26

Table S5. Changes in OFP between leaves of different ages for *Ginkgo biloba*, *Ligustrum lucidum*, and *Forsythia suspensa* (Compared with the previous growth stage) (Units: %).

Plant	Compound	Young leaves	Mature leaves	Senescent leaves
Ginkgo biloba	Total VOCs	—	−67.76	−65.28
	Isoprene	—	−4.23	91.10
	Monoterpenes	—	−45.68	−62.51
	Sesquiterpenes	—	−88.47	−96.63
	Other VOCs	—	−92.04	−85.27
Ligustrum lucidum	Total VOCs	—	−62.01	−32.13
	Isoprene	—	−85.55	31.37
	Monoterpenes	—	−43.23	−30.67
	Sesquiterpenes	—	−88.41	−87.55
	Other VOCs	—	−93.03	−10.83
Forsythia suspensa	Total VOCs	—	−50.88	−81.51
	Isoprene	—	−84.46	−42.63
	Monoterpenes	—	−30.65	−78.76
	Sesquiterpenes	—	−81.40	−99.18
	Other VOCs	—	−74.06	−92.74

Table S6. Changes in OFP contributions between leaves of different ages for *Ginkgo biloba*, *Ligustrum lucidum*, and *Forsythia suspensa* (Compared with the previous growth stage) (Units: %).

Plant	Compound	Young leaves	Mature leaves	Senescent leaves
Ginkgo biloba	Isoprene	—	0.35	2.35
	Monoterpenes	—	34.97	6.87
	Sesquiterpenes	—	−8.12	−4.08
	Other VOCs	—	−27.20	−5.14
Ligustrum lucidum	Isoprene	—	−1.30	0.74
	Monoterpenes	—	29.92	1.94
	Sesquiterpenes	—	−10.96	−3.93
	Other VOCs	—	−17.67	1.25
Forsythia suspensa	Isoprene	—	−1.87	1.82
	Monoterpenes	—	23.23	11.86
	Sesquiterpenes	—	−8.56	−4.98
	Other VOCs	—	−12.80	−8.70

Table S7. Changes in SOAP between leaves of different ages for *Ginkgo biloba*, *Ligustrum lucidum*, and *Forsythia suspensa* (Compared with the previous growth stage) (Units: %).

Plant	Compound	Young leaves	Mature leaves	Senescent leaves
Ginkgo biloba	Total VOCs	—	−54.46	−65.24
	Isoprene	—	−4.23	91.10
	Monoterpenes	—	−38.56	−62.47
	Sesquiterpenes	—	−88.47	−96.63
	Other VOCs	—	−84.49	−87.77
Ligustrum lucidum	Total VOCs	—	−52.52	−37.18
	Isoprene	—	−85.55	31.37
	Monoterpenes	—	−35.48	−33.39
	Sesquiterpenes	—	−88.41	−87.55
	Other VOCs	—	−97.54	−15.78
Forsythia suspensa	Total VOCs	—	−40.41	−80.90
	Isoprene	—	−84.46	−42.63
	Monoterpenes	—	−21.67	−79.06
	Sesquiterpenes	—	−81.40	−99.18
	Other VOCs	—	−78.35	−88.56

Table S8. Changes in SOAP contribution between leaves of different ages for *Ginkgo biloba*, *Ligustrum lucidum*, and *Forsythia suspensa* (Compared with the previous growth stage) (Units: %).

Plant	Compound	Young leaves	Mature leaves	Senescent leaves
Ginkgo biloba	Isoprene	—	0.01	0.06
	Monoterpenes	—	23.64	7.27
	Sesquiterpenes	—	−20.24	−6.20
	Other VOCs	—	−3.40	−1.14
Ligustrum lucidum	Isoprene	—	−0.05	0.02
	Monoterpenes	—	24.51	5.59
	Sesquiterpenes	—	−21.87	−5.66
	Other VOCs	—	−2.59	0.05
Forsythia suspensa	Isoprene	—	−0.07	0.05
	Monoterpenes	—	21.52	8.67
	Sesquiterpenes	—	−18.57	−8.07
	Other VOCs	—	−2.87	−0.66

Disclaimer/Publisher's Note: The statements, opinions and data contained in all publications are solely those of the individual author(s) and contributor(s) and not of MDPI and/or the editor(s). MDPI and/or the editor(s) disclaim responsibility for any injury to people or property resulting from any ideas, methods, instructions or products referred to in the content.
